# Multimodal Computational Approach for Forecasting Cardiovascular Aging Based on Immune and Clinical–Biochemical Parameters

**DOI:** 10.3390/diagnostics15151903

**Published:** 2025-07-29

**Authors:** Madina Suleimenova, Kuat Abzaliyev, Ainur Manapova, Madina Mansurova, Symbat Abzaliyeva, Saule Doskozhayeva, Akbota Bugibayeva, Almagul Kurmanova, Diana Sundetova, Merey Abdykassymova, Ulzhas Sagalbayeva

**Affiliations:** 1 Department of Big Data and Artificial Intelligence, Faculty of Information Technology, Al-Farabi Kazakh National University, Almaty 050040, Kazakhstan; mansurova.madina@gmail.com (M.M.); abzaliyeva.symbat@gmail.com (S.A.); 2 Department of Internal Medicine, Faculty of Medicine and Healthcare, Al-Farabi Kazakh National University, Almaty 050040, Kazakhstan; abzaliev_kuat@mail.ru (K.A.); bota_88.20@mail.ru (A.B.); almagul.kurmanova@kaznu.edu.kz (A.K.); sdiana92@mail.ru (D.S.); merei1808@mail.ru (M.A.); ulya_sagalbayeva@mail.ru (U.S.); 3 Center for Scientific Research and Competence, Civil Aviation Academy, Zakarpatskaya St., 44, Almaty 050039, Kazakhstan; a.manapova@math.kz; 4 LLP “Scientific Research International Institute of Postgraduate Education”, Almaty 050043, Kazakhstan; sdoskojaeva@mail.ru

**Keywords:** biomarkers, cardiovascular aging, machine learning, mathematical modeling, immune aging, prediction

## Abstract

**Background**: This study presents an innovative approach to cardiovascular disease (CVD) risk prediction based on a comprehensive analysis of clinical, immunological and biochemical markers using mathematical modelling and machine learning methods. Baseline data include indices of humoral and cellular immunity (CD59, CD16, IL-10, CD14, CD19, CD8, CD4, etc.), cytokines and markers of cardiovascular disease, inflammatory markers (TNF, GM-CSF, CRP), growth and angiogenesis factors (VEGF, PGF), proteins involved in apoptosis and cytotoxicity (perforin, CD95), as well as indices of liver function, kidney function, oxidative stress and heart failure (albumin, cystatin C, N-terminal pro B-type natriuretic peptide (NT-proBNP), superoxide dismutase (SOD), C-reactive protein (CRP), cholinesterase (ChE), cholesterol, and glomerular filtration rate (GFR)). Clinical and behavioural risk factors were also considered: arterial hypertension (AH), previous myocardial infarction (PICS), aortocoronary bypass surgery (CABG) and/or stenting, coronary heart disease (CHD), atrial fibrillation (AF), atrioventricular block (AB block), and diabetes mellitus (DM), as well as lifestyle (smoking, alcohol consumption, physical activity level), education, and body mass index (BMI). **Methods**: The study included 52 patients aged 65 years and older. Based on the clinical, biochemical and immunological data obtained, a model for predicting the risk of premature cardiovascular aging was developed using mathematical modelling and machine learning methods. The aim of the study was to develop a predictive model allowing for the early detection of predisposition to the development of CVDs and their complications. Numerical methods of mathematical modelling, including Runge–Kutta, Adams–Bashforth and backward-directed Euler methods, were used to solve the prediction problem, which made it possible to describe the dynamics of changes in biomarkers and patients’ condition over time with high accuracy. **Results**: HLA-DR (50%), CD14 (41%) and CD16 (38%) showed the highest association with aging processes. BMI was correlated with placental growth factor (37%). The glomerular filtration rate was positively associated with physical activity (47%), whereas SOD activity was negatively correlated with it (48%), reflecting a decline in antioxidant defence. **Conclusions**: The obtained results allow for improving the accuracy of cardiovascular risk prediction, and form personalised recommendations for the prevention and correction of its development.

## 1. Introduction

Circulatory diseases, despite significant progress in diagnosis and therapy, occupy leading positions among all causes of mortality and disability worldwide. According to WHO, they account for more than 30% of all deaths, which emphasises the need for the early detection of risk factors and development of highly accurate predictive models [1].

Traditional approaches to predicting CVD development based on a limited number of biomarkers are often insufficiently sensitive for individual risk assessment, especially in populations with atypical clinical profiles [2]. In this regard, there is an increasing interest in integrative methods of analysis, which makes it possible to consider the systemic nature of CVD pathogenesis [3,4].

Immune markers such as CD16, CD56, IL-10, TNF-α, and cytotoxic proteins (perforin, CD95) play a key role in the development of chronic inflammation, endothelial dysfunction, and atherogenesis [5,6]. Along with this, proteins of metabolic and vascular profile—albumin, NT-proBNP, VEGF, cystatin C—are increasingly considered the predictors of cardiovascular complications [7,8]. Behavioural and social factors—smoking, physical activity, education level—also have a significant impact on cardiovascular health [9].

The development of mathematical models that take into account the multivariate relationships between these variables are of particular value for describing the dynamics of biomarkers and physiological processes. In the present study, methods such as the Runge–Kutta method, Adams–Bashforth method and inverse Euler method were used, which allowed for a more accurate approximation of parameter changes over time and took into account individual characteristics of patients [10,11].

Thus, the aim of the present work was to develop and validate a mathematical model for predicting cardiovascular diseases based on complex analysis of clinical, immunological and biochemical parameters using numerical methods and machine learning algorithms.

Biological age (BA) is currently considered a more accurate indicator of aging processes compared to chronological age [12]. However, there is still no universal approach or a set of indicators that allow for a reliable and reproducible assessment of the rate of biological aging in different populations.

Libert et al. developed a model that predicts a person’s biological age based on physiological measures such as pressure, strength and response from UK Biobank data. Despite limitations due to the cross-sectional nature of the data and the assumption of a constant aging rate, the results were validated on a subset of participants with repeated measurements [13]. The method was found to be robust and can be used to estimate biological age and identify factors that can delay aging.

Mathematical modelling has shown the influence of behavioural and clinical factors on the risk of heart disease in the elderly [14]. The use of Runge–Kutta, Adams–Bashforth and inverse Euler methods to describe the dynamics of risk factors allowed us to develop a model that showed high prognostic accuracy [15]. The presented model can be a useful tool for developing personalised prevention and treatment strategies for the elderly and increasing active life expectancy.

An actual direction in the study of age-related changes in the immune system is the study of the phenomenon of immune senescence (immunosenescence). The unique age-associated immune profile is characteristic of immunotypes which better reflect individual features of the immune system than chronological age and have high stability throughout the year [16]. Thus, the application of a mathematical model demonstrated high sensitivity, autoantibodies to troponin I (cTnI), alpha-actin (ACTC1) and beta-myosin (MYH7B) at the preclinical stages of myocardial damage. In contrast to traditional markers, which are informative at the stage of irreversible changes in cardiomyocytes [17], these cardiac-specific immunoglobulins represent promising biomarkers for the early detection of cardiac changes. Their introduction into clinical practice requires the standardisation of protocols and further studies on large samples.

Metabolic disorders also contribute to premature aging. According to a mathematical model based on biochemical parameters, analysis of blood mononuclear cells 196 CCS from patients who had undergone cancer in childhood showed that the metabolic age of CCS cells exceeded their actual age [18]. These results indicate possible targets for pharmacological interventions to restore mitochondrial function and prevent age-associated pathologies.

The antioxidant enzyme SOD catalyses the conversion of superoxide radicals into less harmful molecules, thereby protecting cells from oxidative damage and premature aging. In a study by Mao C. et al., it was shown that SOD activity can serve as a prognostic biomarker of mortality in elderly women [19]. Further studies are needed to fully understand how SOD activity correlates with aging, and determine its potential as a reliable biomarker of aging and age-related diseases.

Another biomarker traditionally used in the assessment of renal function, low-molecular-weight protein cystatin C, has an interesting potential as a predictor of aging. For example, individuals with high levels of cystatin C had a 39% increased risk of developing cognitive or physical impairment compared to those with normal or low levels. And higher levels of cystatin C, even with relatively normal renal function, are associated with early aging [20]. A positive and non-linear relationship between albumin-to-globulin ratio and cognitive function in elderly Americans has been found. Maintaining the albumin-to-globulin ratio within the normal range may be one of the therapeutic strategies to limit the progression of cognitive impairment in older adults [21]. Recent studies have also shown the potential of NT-proBNP, which is traditionally used to diagnose and assess the severity of heart failure. The presented data suggest that it can be used for prognostic purposes or as a surrogate endpoint in epidemiological studies and interventional studies as the most accurate marker of biological age. However, it should be considered that its level is influenced by both aging and cardiovascular health [22]. Thus, despite the promising data on the prognostic significance of the above biomarkers as potential predictors of aging, additional studies are required to determine their reliability.

Mohamed Z. Sayed-Ahmed examines the integration of mathematical modelling and deep learning (CNN, RNN) techniques to improve the accuracy of cardiovascular disease (CVD) prediction in his paper [23]. A multi-stage approach is proposed: first differential models are used, then deep learning is applied to extract features from large datasets. The results show that the combined approach significantly outperformed traditional machine learning methods, especially due to the consideration of temporal dynamics and the ability to detect hidden patterns in physiological data. A similar approach was used to develop high-fidelity artificial intelligence (AI) to predict cardiovascular aging in individuals over 60 years of age. The model built using the XGBoost algorithm proved to be the most effective, showing an accuracy of 91% and an AUC value of 0.8333, outperforming classical statistical methods such as logistic regression and the k-nearest neighbours (k-NN) algorithm. The model allows us to assess the risks of premature aging by considering key immunomarkers, and clinical and behavioural indicators [24]. Abhishek et al., using data mining techniques and machine learning algorithms to predict CVD risk from medical records, identified significant patterns. Special attention was given to the comparison of different methods of missing data imputation and model building using CatBoost algorithm. The developed model achieved an accuracy of 91% on a Hungarian dataset [25]. The results obtained confirm the potential of machine learning to improve diagnostic accuracy and prognostic approaches.

Thus, the development of mathematical models that consider multivariate relationships between different variables opens new opportunities in CVD risk prediction. Numerical methods used to describe the dynamics of biomarkers and physiological processes are of value.

## 2. Materials and Methods

This study was conducted within the framework of the scientific and technical project No. AP19677754 ‘Development of markers and diagnostic algorithm for detection and prevention of early cardiovascular aging’, State Institution “Ministry of Science and Higher Education of the RK”.

The study included 52 participants aged 60 years and older who were under observation at polyclinics and medical centres of Almaty city. The sex composition of the sample included 18 men (35%) and 34 women (65%). The mean age of the men was 82.9 ± 10.0 years and of the women 81.5 ± 10.0 years.

Participants were divided into two groups according to the presence of cardiovascular disease. The first group included 30 people with an established history of CVD, group 2–22 people without heart and vascular diseases.

Exclusion criteria: HIV infection, known tuberculosis, acute infectious diseases within 3 months prior to inclusion, mental illness that limits adequate co-operation, diagnosed allergic reaction of any type, refusal to participate in the study.

All participants were adequately informed about the aims, methods and conditions of the study, and then gave voluntary and informed consent to participate.

For the analysis of immunological parameters, 50 μL venous blood from patients was collected and transported to the Scientific Centre of Obstetrics, Gynecology and Perinatology in Almaty, Kazakhstan within 2 h. Samples were stained with monoclonal antibodies (mAb) manufactured by Becton Dickinson (BD) (Franklin Lakes, NJ, USA), directed against CD4, CD8, CD16, CD56, CD14, CD19, CD95, and HLA-DR^+^ to detect cell surface receptors. Then, 5 μL of antibodies was added to the tube, mixed on a vortex mixer, and incubated for 15 min at room temperature in the dark. After incubation, lysis was performed using BD FACS Lyse Lysing Buffer, and incubated for 10 min at room temperature. The samples were centrifuged for 5 min at 300× *g* and the supernatant was removed. Next, membrane permeabilization was performed using a Cytofix/Cytoperm solution, and intracellular receptor staining was performed using antibodies against TNF, GM-CSF, VEGFR-2, IPGF and Perforin. The study of immune status was performed on a BD FACS CALIBUR flow cytometer (San Jose, CA, USA) using CELL Quest Pro 4.0 software.

For biochemical analysis, the patients’ venous blood in the volume of 3–5 mL was collected in vacuum tubes without anticoagulant and transported to the clinical diagnostics laboratory within 2 h. Biochemical parameters were determined using an automatic biochemical analyser (Roche Diagnostics, Basel, Switzerland), according to the manufacturer’s instructions. Commercial kits were used as reagents, including those for the determination of albumin, C-reactive protein (CRP), pronatriuretic peptide (NT-pro-BNP), serum cholinesterase, cystatin C, and superoxide dismutase levels. The obtained data were recorded in an electronic laboratory system and used for further statistical analysis.

Problem statement: Based on the obtained clinical and laboratory data, a mathematical model for predicting premature cardiovascular aging was developed. It was based on numerical methods of solving differential equations: the Runge–Kutta method, Adams–Bashforth method and inverse Euler method. Pearson correlation analysis was used to identify the relationships between variables. The model describes the dynamic interaction of immunological, biochemical and behavioural risk factors over time. And machine learning methods were applied not only to build prognostic models, but also to visualise the relationships between clinical, immunological and behavioural parameters.

The weighting of factors was based on a combination of statistical analysis and machine learning techniques. Pearson’s correlation coefficients were used to assess the strength of association between individual factors and cardiovascular aging. In addition, numerical modelling using the Runge–Kutta, Adams–Bashforth, and backward Euler methods enabled the simulation of dynamic interactions among risk factors over time.

As a result, the selected biomarkers captured the core mechanisms underlying cardiovascular aging, and their contributions to the model were determined through an evidence-based and comprehensive analytical approach. This ensured both high predictive accuracy and the potential for personalised preventive strategies.

A machine learning (ML) framework was developed to predict the rate of aging and the risk of cardiovascular disease (CVD), incorporating algorithms such as random forest, logistic regression, k-nearest neighbours and XGBoost [24].

Table 1, Table 2 and Table 3 present the definitions of immunological, biochemical biomarkers and socio-behavioural factors.

The selection of immunological, biochemical and socio-behavioural parameters was guided by evidence from peer-reviewed literature and expert consultation. Biomarkers such as *CD14*, *CD16*, *HLA-DR*, *CRP*, *SOD* and *cystatin C* have been repeatedly shown to be associated with inflammation, oxidative stress, immune dysregulation and cardiac remodelling, all central to cardiovascular aging [5,6,7,8,16,17,18,19,20,21,22].

Social and behavioural factors, including smoking (*S*), alcohol consumption (*A*), physical activity (*PhA*), education (*E*) and *BMI*, were included based on their well-established associations with cardiovascular outcomes in large epidemiological studies, such as Framingham, NHANES and WHO global CVD reports [1,9].

Together, these features represent key physiological and behavioural determinants of biological and cardiovascular aging, and ensure that the model captures both internal biological processes and modifiable external risk factors.

We considered 2 experimental datasets, immunological (Problem 1) and biochemical (Problem 2) biomarkers, to predict cardiovascular aging using a mathematical model. Each set is represented by 3 cases: biomarkers of patients without a CVD diagnosis (Case A), biomarkers of patients with a CVD diagnosis (Case B) and biomarkers of patients without the presence of a CVD diagnosis (Case C).

Pearson correlation was used to calculate the correlation coefficients of the interaction between biomarkers (immunological (Problem 1) and biochemical (Problem 2)) and clinical parameters.

A mathematical model is created taking into account the level of interaction between medical biomarkers (Problem 1 and Problem 2) and clinical parameters:

(1) $$A^{\prime }=\sum_{i=1}^{6}a_{i}x_{i}y_{i},$$

where $A^{\prime }$ is derivative, responsible for changes in aging over time t, $i=\bar{1\ldots 6}$, coefficient $a_{i}$ is the Pearson correlation coefficient, $x_{i}$ is the immunological biomarker and $y_{i}$ is the social parameter.

**Figure 1:**
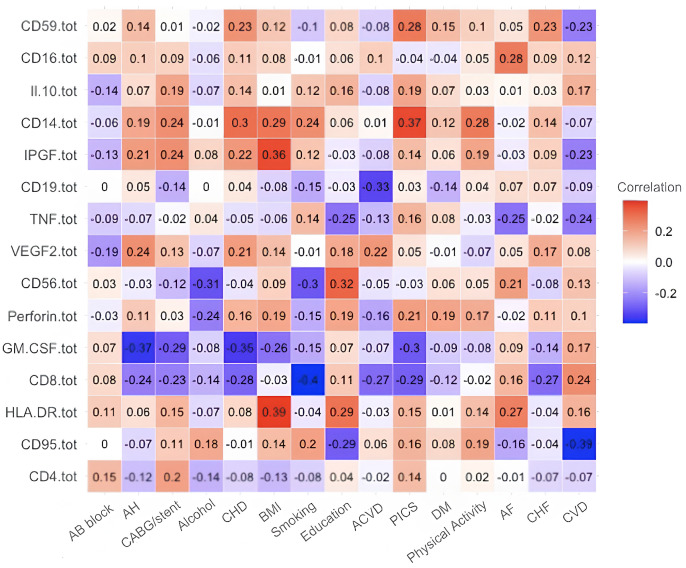
Correlation coefficient of the immunological biomarkers of all experimental patients (Case A of Problem 1).

Figure 1 shows the results of the correlation coefficient by immunological biomarkers for all experimental patients. High levels of interaction between body mass index parameters (hereinafter BMI) and major histocompatibility complex class II molecule (hereinafter HLA-DR) are indicated by interaction coefficient $a_{1}$, postinfarction cardiosclerosis (hereinafter PICS) and receptor found on the surface of monocytes and macrophages (hereinafter CD14) with interaction coefficient $b_{1}$, BMI and placental growth factor associated with vascular development (IPGF) with interaction coefficient $c_{1}$, education (hereinafter E) and a protein that attaches to the membrane of cells and protects them from damage by the immune system by preventing complement activation (hereinafter CD59) with interaction factor $d_{1}$, BMI and CD14 with interaction factor $e_{1}$, and coronary heart disease (hereinafter CHD) and CD14 with interaction factor $f_{1}$.

From Figure 1, high correlation coefficients are picked up and presented in Table 4.

For Equation (1), medical biomarkers and socio-behavioural factors were obtained from correlation analysis and are presented in Table 5.

The definitions of the presented parameters of Table 5 are explained in Table 1 and Table 3.

Let the following notations be used in the following model: *E*—level of education (socio-behavioural factor), *P*—physical activity level, *S*—smoking status, *A*—alcohol consumption level, *C*ℎ—serum cholinesterase level (ChE), etc. (see Table 3).

Then, we transform Equation (1) in the form of (2) to construct a mathematical model of premature aging taking into account Table 5 in the form of a differential equation:

(2) $$\begin{array}{c} \frac{dA}{dt}=a_{1}\cdot \mathrm{BMI}\cdot \mathrm{HLA}.\mathrm{DR}+b_{1}\cdot \mathrm{PICS}\cdot \mathrm{CD}14+c_{1}\cdot \mathrm{BMI}\cdot \mathrm{IPGF}+d_{1}\cdot E\cdot \mathrm{CD}59\\ +e_{1}\cdot \mathrm{BMI}\cdot \mathrm{CD}14+f_{1}\cdot \mathrm{CHD}\cdot \mathrm{CD}14 \end{array}$$

where on the right-hand side, (2) $\frac{dA}{dt}$ means changes in premature aging over time, where $\frac{d}{dt}$ is a differential operator, A is aging and t is time. And in the left part of (1), each summand represents the interactions of social and immunological parameters with the level of interaction, which has the value of correlation coefficient, and the sum of summands means that premature aging is affected by the interactions of not only two parameters. From (1), we can conclude that changes in the major histocompatibility complex class II molecule, placental growth factor associated with vascular development and the receptor found on the surface of monocytes and macrophages cause abnormalities in the body mass index. Additionally, the receptor found on the surface of monocytes and macrophages affects the occurrence of postinfarction cardiosclerosis and coronary heart disease. And, we can pay attention to a very interesting fact that there is a 32% probability that the presence of the patient’s education interacts with a protein that attaches to the membrane of cells and protects them from damage by the immune system by preventing complement activation. This can be attributed to the fact that the human gene product has a direct effect on the level of education of the individual.

Equation (2) expresses the same model in an expanded differential form, where each term represents the interaction between a selected biomarker and a socio-behavioural factor weighted by its correlation coefficient.

**Figure 2:**
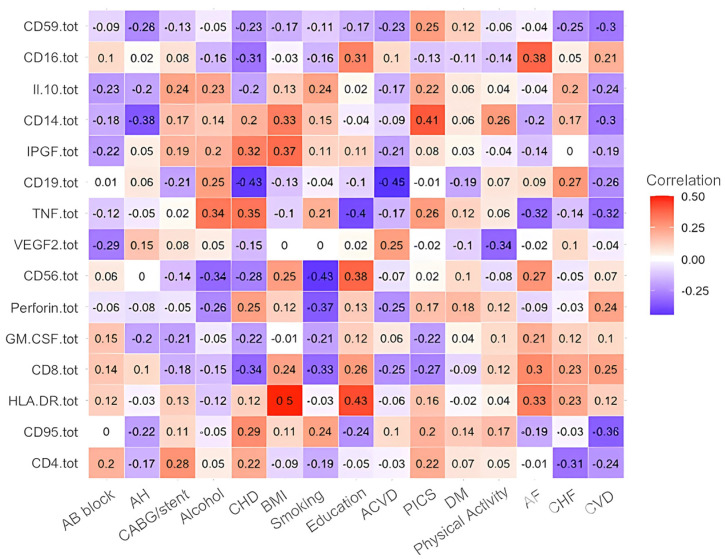
Correlation coefficient of the immunological biomarkers of patients with CVD (Case B of Problem 1).

**Figure 3:**
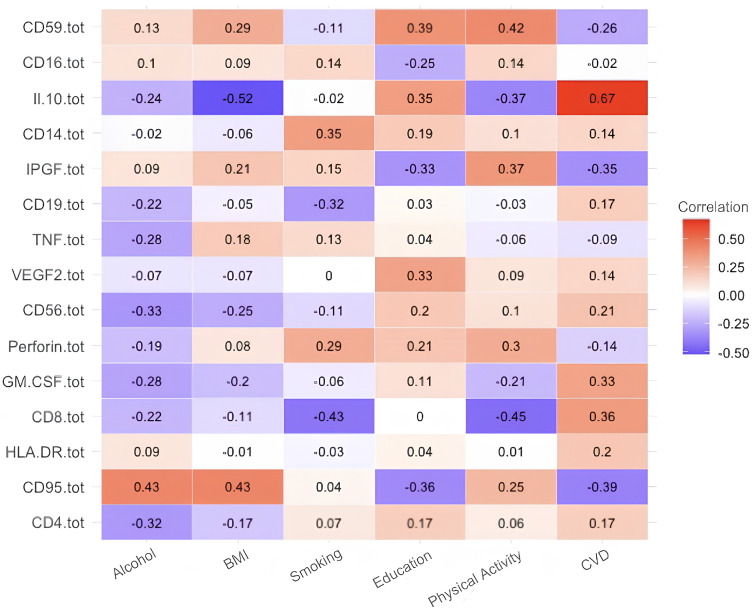
Correlation coefficient of the immunological biomarkers of patients without CVD (Case C of Problem 1).

**Figure 4:**
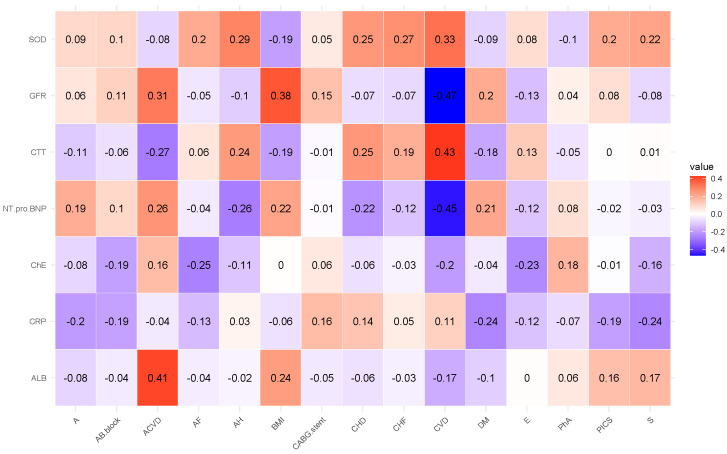
Correlation coefficient of the biochemical biomarkers of all experimented patients (Case A of Problem 2).

**Figure 5:**
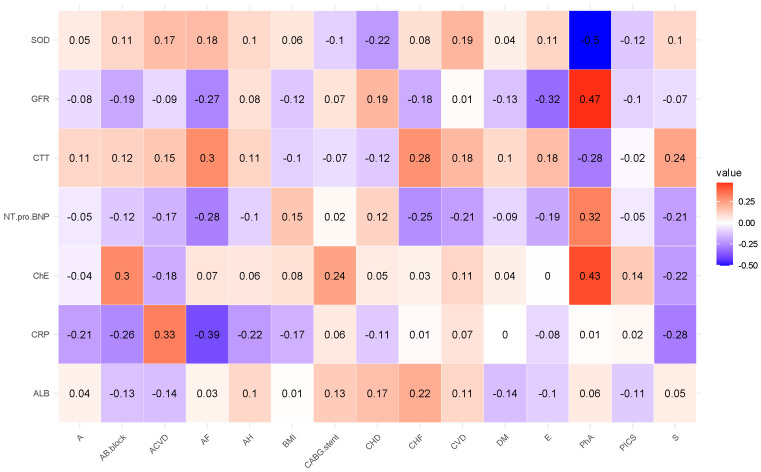
Correlation coefficient of the biochemical biomarkers of patients with CVD (Case B of Problem 2).

**Figure 6:**
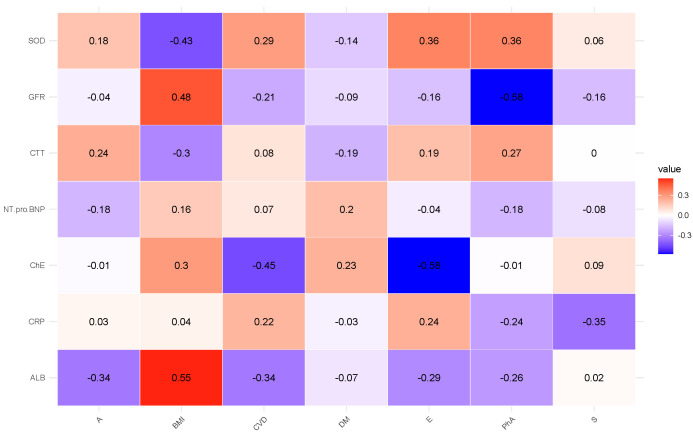
Correlation coefficient of the biochemical biomarkers of patients without CVD (Case C of Problem 2).

Next, in this order, we present the correlation analyses of the remaining studied case in Figure 2, Figure 3, Figure 4, Figure 5 and Figure 6 and Table 6, Table 7, Table 8, Table 9, Table 10, Table 11, Table 12, Table 13, Table 14 and Table 15 [Table 6, Table 8, Table 10, Table 12 and Table 14 of high-interaction coefficients between medical biomarkers and socio-behavioural factors, and their definitions (Table 7, Table 9, Table 11, Table 13 and Table 15)].

Now, we numerically solve Equation (2) by Runge-Kutta methods of order 4, Adams–Bashforth and inverse Euler. Let us first present the application of the first method to mathematical model (2).

The desired variable is represented in the following form:

(3) $$A_{n+1}=\;A_{n}+\;\frac{h}{6}\cdot (k_{1}+2k_{2}+2k_{3}+k_{4}),$$

where *h* = $\frac{b-a}{N}$ is the computational step, and $k_{1}$, $k_{2}$, $k_{3}$, $k_{4}$ are the method coefficients.

$$k_{1}=f\left(x_{n},t_{n}\right),$$

$$k_{2}=f\left(t_{n}+\frac{h}{2},x_{n}+\frac{h}{2}k_{1}\right),$$

$$k_{3}=f\left(t_{n}+\frac{h}{2},x_{n}+\frac{h}{2}k_{2}\right),$$

$$k_{4}=f\left(t_{n}+h,x_{n}+hk_{3}\right).$$

In our case, the method coefficients will take the following form:

$$\begin{array}{c} k_{1}=a_{1}\cdot \mathrm{BMI}\left(x_{n},\;t_{n}\right)\cdot HLA.DR\left(x_{n},\;t_{n}\right)+b_{1}\cdot PICS\left(x_{n},\;t_{n}\right)\cdot \mathrm{CD}14\left(x_{n},\;t_{n}\right)+c_{1}\cdot \mathrm{BMI}\left(x_{n},\;t_{n}\right)\cdot \\ IPGF\left(x_{n},\;t_{n}\right){+d}_{1}\cdot E\cdot \mathrm{CD}59\left(x_{n},\;t_{n}\right)+e_{1}\cdot \mathrm{BMI}\left(x_{n},\;t_{n}\right)\cdot \mathrm{CD}14\left(x_{n},\;t_{n}\right)+f_{1}\cdot \mathrm{CHD}\left(x_{n},\;t_{n}\right)\cdot \mathrm{CD}14\left(x_{n},\;t_{n}\right), \end{array}$$

$$\begin{array}{c} k_{2}=a_{1}\cdot \mathrm{BMI}\left(t_{n}+\frac{h}{2},\;x_{n}+\frac{h}{2}k_{1}\right)\cdot HLA.DR\left(t_{n}+\frac{h}{2},\;x_{n}+\frac{h}{2}k_{1}\right)+b_{1}\cdot PICS\left(t_{n}+\frac{h}{2},\;x_{n}+\frac{h}{2}k_{1}\right)\cdot \mathrm{CD}14(t_{n}+\frac{h}{2},\;x_{n}+\\ \frac{h}{2}k_{1})+c_{1}\cdot \mathrm{BMI}\left(t_{n}+\frac{h}{2},\;x_{n}+\frac{h}{2}k_{1}\right)\cdot IPGF\left(t_{n}+\frac{h}{2},\;x_{n}+\frac{h}{2}k_{1}\right){+d}_{1}\cdot E\left(t_{n}+\frac{h}{2},\;x_{n}+\frac{h}{2}k_{1}\right)\cdot \mathrm{CD}59\left(t_{n}+\frac{h}{2},\;x_{n}+\frac{h}{2}k_{1}\right)+\\ e_{1}\cdot \mathrm{BMI}\left(t_{n}+\frac{h}{2},\;x_{n}+\frac{h}{2}k_{1}\right)\cdot \mathrm{CD}14\left(t_{n}+\frac{h}{2},\;x_{n}+\frac{h}{2}k_{1}\right)+f_{1}\cdot \mathrm{CHD}\left(t_{n}+\frac{h}{2},\;x_{n}+\frac{h}{2}k_{1}\right)\cdot \mathrm{CD}14\left(t_{n}+\frac{h}{2},\;x_{n}+\frac{h}{2}k_{1}\right), \end{array}$$

$$\begin{array}{c} k_{3}=a_{1}\cdot \mathrm{BMI}\left(t_{n}+\frac{h}{2},\;x_{n}+\frac{h}{2}k_{2}\right)\cdot HLA.DR\left(t_{n}+\frac{h}{2},\;x_{n}+\frac{h}{2}k_{2}\right)+b_{1}\cdot PICS\left(t_{n}+\frac{h}{2},\;x_{n}+\frac{h}{2}k_{2}\right)\cdot \mathrm{CD}14(t_{n}+\frac{h}{2},\;x_{n}+\\ \frac{h}{2}k_{2})+c_{1}\cdot \mathrm{BMI}\left(t_{n}+\frac{h}{2},\;x_{n}+\frac{h}{2}k_{2}\right)\cdot IPGF\left(t_{n}+\frac{h}{2},\;x_{n}+\frac{h}{2}k_{2}\right){+d}_{1}\cdot E\left(t_{n}+\frac{h}{2},\;x_{n}+\frac{h}{2}k_{2}\right)\cdot \mathrm{CD}59\left(t_{n}+\frac{h}{2},\;x_{n}+\frac{h}{2}k_{2}\right)+\\ e_{1}\cdot \mathrm{BMI}\left(t_{n}+\frac{h}{2},\;x_{n}+\frac{h}{2}k_{2}\right)\cdot \mathrm{CD}14\left(t_{n}+\frac{h}{2},\;x_{n}+\frac{h}{2}k_{2}\right)+f_{1}\cdot \mathrm{CHD}\left(t_{n}+\frac{h}{2},\;x_{n}+\frac{h}{2}k_{2}\right)\cdot \mathrm{CD}14\left(t_{n}+\frac{h}{2},\;x_{n}+\frac{h}{2}k_{2}\right), \end{array}$$

$$\begin{array}{c} k_{4}=a_{1}\cdot \mathrm{BMI}\left(t_{n}+h,\;x_{n}+hk_{3}\right)\cdot HLA.DR\left(t_{n}+h,\;x_{n}+hk_{3}\right)+b_{1}\cdot PICS\left(t_{n}+h,\;x_{n}+hk_{3}\right)\cdot \mathrm{CD}14\left(t_{n}+h,\;x_{n}+hk_{3}\right)+\\ c_{1}\cdot \mathrm{BMI}\left(t_{n}+h,\;x_{n}+hk_{3}\right)\cdot IPGF\left(t_{n}+h,\;x_{n}+hk_{3}\right){+d}_{1}\cdot E\left(t_{n}+h,\;x_{n}+hk_{3}\right)\cdot \mathrm{CD}59\left(t_{n}+h,\;x_{n}+hk_{3}\right)+e_{1}\cdot \\ \mathrm{BMI}\left(t_{n}+h,\;x_{n}+hk_{3}\right)\cdot \mathrm{CD}14\left(t_{n}+h,\;x_{n}+hk_{3}\right)+f_{1}\cdot \mathrm{CHD}\left(t_{n}+h,\;x_{n}+hk_{3}\right)\cdot \mathrm{CD}14\left(t_{n}+h,\;x_{n}+hk_{3}\right). \end{array}$$

We considered the application of the following second-order Adams–Bashforth method to compare the numerical results:

(4) $$A_{i+1}=A_{i}+\frac{3}{2}\cdot ∆t\cdot {\mathrm{RHS}}_{i}-\frac{1}{2}\cdot ∆t\cdot {\mathrm{RHS}}_{i-1}$$

where $A_{i}$ is the current aging value, ${\mathrm{RHS}}_{i}$ is the right-hand side of Equation (2), ${\mathrm{RHS}}_{i-1}$ is the right-hand side of Equation (2) at the previous point, and ∆t is the time step.

The third backward Euler method was used, which can simplify the calculation time and is a less costly method:

(5) $$A_{i+1}=A_{i}+∆t\cdot {\mathrm{RHS}}_{i+1}$$

where ∆t is the time step, and ${\mathrm{RHS}}_{i+1}$ is the right-hand side of Equation (2) at a new point.

We selected the fourth-order Runge–Kutta method due to its balance between computational efficiency and high accuracy. It is particularly well-suited for solving nonlinear ordinary differential equations (ODEs) that arise in modelling biological processes, as it provides the global error of order (*h*^4^) and maintains numerical stability over relatively large steps.

The Adams–Bashforth method, being a multistep explicit technique, was chosen to examine how the use of historical derivative information (from previous time steps) influences the accuracy of the predictions. This is relevant to biological systems that exhibit inertia or memory effects.

The backward Euler method was included as a simple, first-order implicit scheme, primarily for comparative purposes. Despite its lower accuracy (*h*), it is known for strong numerical stability and is often used for stiff equations.

The comparative analysis (Table 16 and Table 17) clearly demonstrates that the Runge–Kutta method outperforms the other two in terms of maximum absolute error across all case types. This result supports our decision to adopt Runge–Kutta for the final implementation of the mathematical model.

To verify the numerical methods, it was necessary to obtain the analytical (exact) solutions of Equation (2). In order to avoid the differential, let us integrate Equation (2) in time from both sides:

(6) $$\begin{array}{c} A={t\cdot a}_{1}\cdot {\mathrm{BMI}}_{i}\cdot {\mathrm{HLA}.\mathrm{DR}}_{i}+{t\cdot b}_{1}\cdot {\mathrm{PICS}}_{i}\cdot {\mathrm{CD}14}_{i}+{t\cdot c}_{1}\cdot {\mathrm{BMI}}_{i}\cdot {IPGF}_{i}+{t\cdot d}_{1}\cdot E_{i}\cdot {\mathrm{CD}59}_{i}+{t\cdot e}_{1}\cdot {\mathrm{BMI}}_{i}\cdot \\ {\mathrm{CD}14}_{i}+{t\cdot f}_{1}\cdot {CHD}_{i}\cdot {\mathrm{CD}14}_{i} \end{array}$$

The obtained Equation (6) is an exact (analytical) solution of Equation (2). Let us write Equation (6) in finite-difference form for numerical implementation:

(7) $$\begin{array}{c} x^{n+1}=∆t\cdot n\cdot (a_{1}\cdot {\mathrm{BMI}}_{i}\cdot {HLA.DR}_{i}+b_{1}\cdot {PICS}_{i}\cdot {\mathrm{CD}14}_{i}+c_{1}\cdot {\mathrm{BMI}}_{i}\cdot {IPGF}_{i}+d_{1}\cdot E_{i}\cdot {\mathrm{RHS}59}_{i}+e_{1}\cdot {\mathrm{BMI}}_{i}\cdot \\ {\mathrm{CD}14}_{i}+f_{1}\cdot {CHD}_{i}\cdot {\mathrm{CD}14}_{i}) \end{array}$$

All these methods were used for Equations (2) and (3) in the same way as for Equation (4).

We selected the time step in the interval 0<∆t<1, with ∆t=0.01. The grid step was $∆x=\frac{1-0}{100}=0.01$.

With the above, numerical results could be described and real-time analyses could be conducted with N·∆t=t, where N=100 was the number of iterations in the program code.

The created mathematical models from Equations (2)–(4) can be considered experiments for future similar studies.

The data from Table 18 were used for the simulation of the numerical solution.

The parameters $Px\left(x,t\right)$, $A\left(x,t\right)$, $I\left(x,t\right)$, $AH\left(x,t\right)$, $S\left(x,t\right)$, $X\left(x,t\right)$, $G\left(x,t\right)$, $ch\left(x,t\right)$ and $E\left(x,t\right)$ used for calculating the correlation coefficient were read from the statistical database. The initial conditions were as follows:

$$\begin{array}{c} Px\left(x,0\right)=0,\;A\left(x,0\right)=0,\;I\left(x,0\right)=0,\;AH\left(x,0\right)=0,\;S\left(x,0\right)=0,\;\\ X\left(x,0\right)=0,\;G\left(x,0\right)=0,\;ch\left(x,0\right)=0,\;E\left(x,0\right)=0 \end{array}$$

The first boundary conditions were as follows:

$$\begin{array}{c} Px\left(0,t\right)=1,\;A\left(0,t\right)=1,\;I\left(0,t\right)=1,\;AH\left(0,t\right)=1,\;S\left(0,t\right)=1,\;\\ X\left(0,t\right)=1,\;G\left(0,t\right)=1,\;ch\left(0,t\right)=1,\;E\left(0,t\right)=1 \end{array}$$

For the numerical solution of the equations, these parameters have the following expressions:

$$\mathrm{BMI}\left(x,t\right)=IMT\left(x-1,t\right)+i∆x,$$

$$PICS\left(x,t\right)=PICS\left(x-1,t\right)+i∆x,$$

$$E\left(x,t\right)=E\left(x-1,t\right)+i∆x,$$

$$\mathrm{CHD}\left(x,t\right)=IBS\left(x-1,t\right)+i∆x,$$

$$HLA.DR\left(x,t\right)=HLA.DR\left(x-1,t\right)+i∆x,$$

$$\mathrm{CD}14\left(x,t\right)=\mathrm{CD}14\left(x-1,t\right)+i∆x,$$

$$IPGF\left(x,t\right)=IPGF\left(x-1,t\right)+i∆x,$$

$$\mathrm{CD}59\left(x,t\right)=\mathrm{CD}59\left(x-1,t\right)+i∆x,$$

$$AH\left(x,t\right)=AH\left(x-1,t\right)+i∆x,$$

$$\mathrm{CD}56\left(x,t\right)=\mathrm{CD}56\left(x-1,t\right)+i∆x,$$

$$\mathrm{CD}16\left(x,t\right)=\mathrm{CD}16\left(x-1,t\right)+i∆x,$$

$$CVD\left(x,t\right)=CVD\left(x-1,t\right)+i∆x,$$

$$A\left(x,t\right)=A\left(x-1,t\right)+i∆x,$$

$$PhA\left(x,t\right)=PhA\left(x-1,t\right)+i∆x,$$

$$II10\left(x,t\right)=II10\left(x-1,t\right)+i∆x,$$

$$\mathrm{CD}95\left(x,t\right)=\mathrm{CD}95\left(x-1,t\right)+i∆x,$$

$$ACVD\left(x,t\right)=ACVD\left(x-1,t\right)+i∆x,$$

$$CTT\left(x,t\right)=CTT\left(x-1,t\right)+i∆x,$$

$$ALB\left(x,t\right)=ALB\left(x-1,t\right)+i∆x,$$

$$GFR\left(x,t\right)=GFR\left(x-1,t\right)+i∆x,$$

$$SOD\left(x,t\right)=SOD\left(x-1,t\right)+i∆x,$$

$$AB\left(x,t\right)=AB\left(x-1,t\right)+i∆x,$$

$$S\left(x,t\right)=S\left(x-1,t\right)+i∆x,$$

$$stent\left(x,t\right)=stent\left(x-1,t\right)+i∆x,$$

$$ChE\left(x,t\right)=ChE\left(x-1,t\right)+i∆x,$$

$$CRP\left(x,t\right)=CRP\left(x-1,t\right)+i∆x,$$

here, $∆x=\frac{b-a}{N}$ is the grid spacing. However, in order to obtain previous values, the parameters had their values taken from the statistical database.

## 3. Results

**Figure 7:**
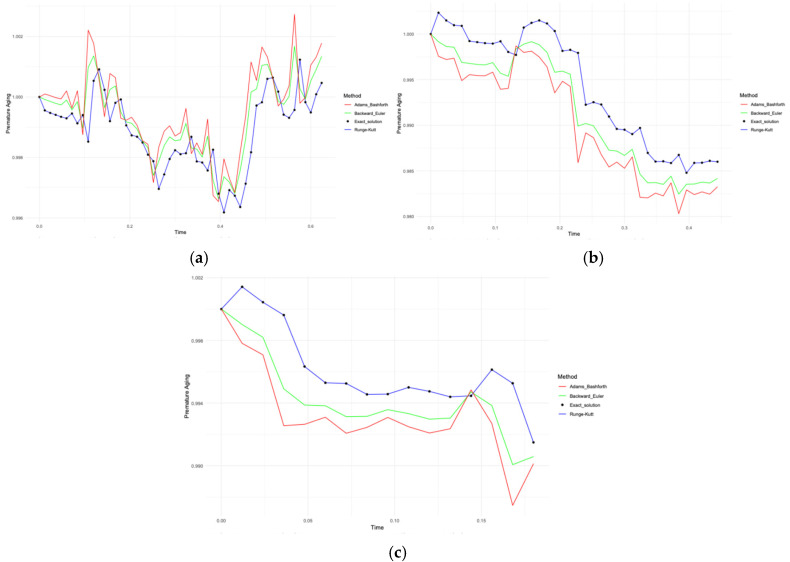
(**a**) Comparison of numerical methods for modelling premature aging with immunological biomarkers for all patients (Case A of Problem 1); (**b**) for patients with CVD (Case B of Problem 1); and (**c**) for patients without CVD (Case C of Problem 1).

Figure 7a–c shows the changes in aging by the interaction of immunological biomarkers with socio-behavioural factors by the Runge–Kutta, Adams–Bashforth and inverse Euler methods compared to the exact solution.

Figure 7a–c show the behaviour of immunological biomarker-based premature aging models for three cases: all patients (Case A), patients with CVD (Case B) and patients without CVD (Case C). All plots include a comparison of the numerical methods Runge–Kutta, Adams–Bashforth, inverse Euler and exact solution. In Case A (Figure 7a), the Runge–Kutta method matched almost perfectly with the analytical solution (maximum error 0.000007536924), whereas the inverse Euler and Adams–Bashforth methods had more pronounced deviations (0.00424 and 0.00636, respectively). In Case B (Figure 7b), a similar pattern was observed:: the minimum error for Runge–Kutta was 0.000004918764 in contrast to 0.00304603 and 0.004569044. In Case C (Figure 7c), again, the Runge–Kutta method showed the smallest error of 0.00000284855.

Differences between the numerical and exact solutions were found (Table 16).

**Figure 8:**
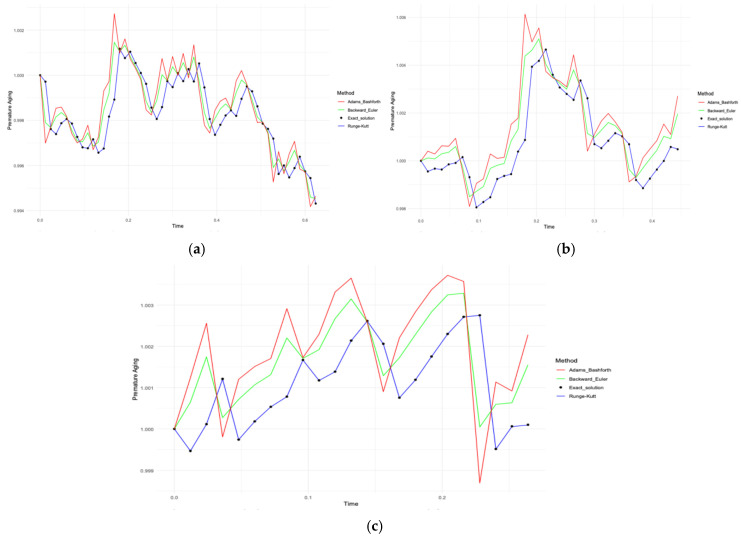
(**a**) Comparison of numerical methods for modelling premature aging with biochemical biomarkers for all patients (Case A of Problem 2); (**b**) for patients with CVD (Case B of Problem 2); and (**c**) for patients without CVD (Case C of Problem 2).

Figure 8a–c similarly illustrates the modelling results for biochemical biomarkers. For Case A (Figure 8a), the Runge–Kutta error was 0.000004710849; for the inverse Euler method, the error was 0.002740348; and for the Adams–Bashforth method, it was 0.004110521. In Case B (Figure 8b), the Runge-Kutta was 0.00001958067, inverse Euler 0.003500058 and Adams–Bashforth 0.005250088. In Case C (Figure 8c), the Runge–Kutta was 0.000002147958, inverse Euler 0.004239759 and Adams–Bashforth 0.006359639. Thus, the fourth-order Runge–Kutta was the most accurate numerical solution method, regardless of the type of data and the presence of CVD diagnosis.

**Figure 9:**
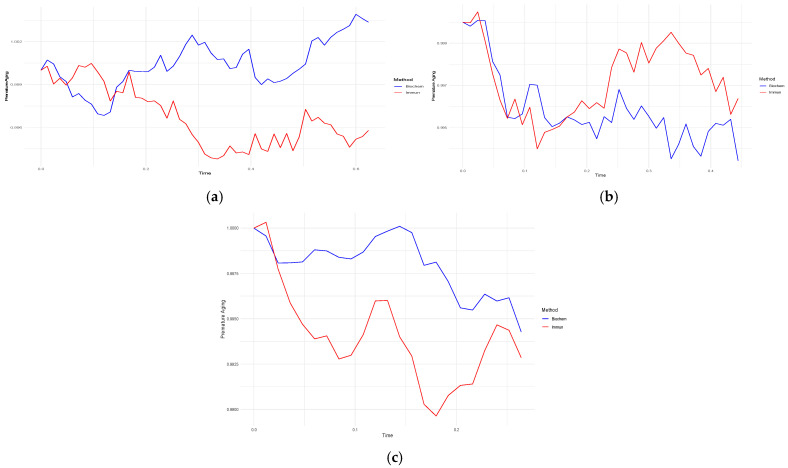
(**a**) Comparison of aging changes in immunological and biochemical biomarkers for all patients according to the Runge–Kutta method; (**b**) for patients with CVDs according to the Runge–Kutta method; and (**c**) for patients without CVDs according to the Runge–Kutta method.

According to the data in Table 16 and Table 17, the Runge–Kutta method of the 4th order showed the lowest error from the exact solution in contrast to the other numerical methods; we stop on this method. Figure 9a–c compares the behaviour of premature aging models based on immunological and biochemical biomarkers with the Runge–Kutta method. In Case A (Figure 9a), biochemical markers showed higher aging values compared to immunological markers, especially late in the timeline. In Case B (Figure 9b), both types of data coincided at the beginning of the time interval, but then immunological markers showed a sharp increase in aging, confirming their key role in the presence of CVD. In Case C (Figure 9c), biochemical markers again dominated the level of aging, especially in the absence of CVD.

Figure 9a–c shows the difference according to the above-mentioned cases (Cases A, B, C). In Figure 9a, one can notice high aging rates of biochemical data compared to immunological data. This makes it clear that without the presence of CVD data, biochemical data, which are not resource-intensive, can be chosen to investigate premature aging.

In Figure 9b, at the beginning of time, the biomarker data show similar results. Over time, a sharp peak in immunological data can be seen, which confirms that the data with CVD are central to the definition of premature aging.

In Figure 9c, where the data without CVD are presented, high rates of biochemical data are noticeable.

As mentioned above, immunological data were obtained in a resource-intensive way, at the same time they presented high values. As a result, it can be concluded that the CVD-adjusted data are the best for determining premature aging with CVD.

## 4. Discussion

The results of the study showed that major histocompatibility complex class II (MHC II) molecules interacting with the body mass index gave the highest level of correlation at 50%, indicating a significant influence of these immunological indicators on biological aging. There was also an interaction of education level with the same MHC II molecules at 43%, which may indicate an underlying relationship between cognitive and immune processes. The CD14 receptor, located on the surface of monocytes and macrophages, showed a 41% level of association with post-infarct cardiosclerosis and 30% with coronary heart disease, emphasising its role in inflammatory and post-necrotic processes. The NK cell marker CD56 correlated with education levels by 38%, and the CD16 receptor present on NK cells and neutrophils showed a similar level of association with blood pressure indices. The immunological indicator IPGF, associated with angiogenesis, had a 37% correlation with body mass index, indicating a vascular component of metabolic disorders. In the biochemical profile, the glomerular filtration rate was positively correlated with the level of physical activity at 47%, while superoxide dismutase showed a negative correlation with the same parameter at 48%, reflecting a decrease in antioxidant activity with increasing metabolic load.

To investigate predictive patterns of premature aging, machine learning models were initially trained using integrated clinical and immunological datasets. Models such as random forest (RF), logistic regression (LR), and k-nearest neighbours (k-NN) achieved a maximum accuracy of 0.81 and an area under the ROC curve (AUC) of 0.69. To enhance predictive performance, the XGBoost algorithm was subsequently employed on the same dataset. This model demonstrated superior results, achieving an accuracy of 91% and an AUC of 0.8333, highlighting its robustness in detecting immunological patterns associated with premature aging.

Prior to model training, extensive data preprocessing was performed. Given the significant class imbalance observed in the dataset, the Synthetic Minority Over-Sampling Technique (SMOTE) was applied to generate synthetic instances of the minority class based on its nearest neighbours. This approach effectively mitigated the model’s bias toward the majority class and contributed to improved generalisation.

Furthermore, in the subsequent stage of the study, a predictive model for cardiovascular disease (CVD) was developed using XGBoost in combination with SMOTE, demonstrating both high accuracy and resilience to feature multicollinearity.

Moreover, an alternative approach to addressing this problem involves the use of machine learning techniques applied to a curated biomarker database. The algorithm and its implementation are described in detail in our previous work [24], offering a complementary computational strategy for the early detection of biological aging processes.

The model has certain limitations. Its performance is influenced by the quality and completeness of the clinical data, which may limit its applicability to other datasets. The selected biomarkers reflect only a subset of the multifactorial biological processes underlying aging. Additionally, since the model was trained on a specific cohort, further validation on larger and more diverse populations is necessary to confirm its generalizability.

In addition to the limitations already noted, the relatively small sample size (*n* = 52) reduces the statistical power and generalizability of the findings. The study was conducted within a single geographic and demographic population, which may introduce bias or limit applicability to broader cohorts. Moreover, the model was trained and tested on the same dataset, without an independent external validation cohort. While internal cross-validation was employed to mitigate overfitting, external validation on diverse populations is essential to confirm robustness and reproducibility.

All participants were recruited from a single urban region in Kazakhstan, resulting in limited ethnic, geographic and socioeconomic diversity. This may restrict the generalizability of the findings to other populations or healthcare settings.

The current model does not explicitly account for potential confounding factors such as genetic predispositions, medication use, hormonal status, psychosocial stress or undiagnosed comorbidities. These variables may influence both biomarker levels and aging outcomes, introducing potential bias.

Traditional cardiovascular risk models, such as the Framingham Risk Score and SCORE, rely primarily on clinical and lifestyle indicators (e.g., blood pressure, cholesterol, smoking), and are limited in their ability to incorporate immune or biochemical markers. These models are often based on logistic regression and assume linear relationships, which may not capture the complex, dynamic nature of aging.

In contrast, more recent approaches, including deep learning models (CNNs, RNNs), showed improved predictive performance but are often criticised for their lack of interpretability. For example, the study by Mohamed Z. Sayed-Ahmed [23] utilised a hybrid model combining differential equations and deep learning for CVD prediction, achieving high accuracy but limited transparency in biomarker contribution.

Furthermore, the mathematical modelling approach is based on several assumptions: (i) linear correlation (via Pearson coefficients) adequately captures the interactions between biomarkers and aging processes, and (ii) the dynamics of aging can be reliably described by ordinary differential equations. These simplifications may not fully reflect the complexity of biological aging, or the possible non-linear and latent interactions between variables. Future work should consider more comprehensive multivariate and dynamic models, as well as integration with external datasets to validate the current findings.

Immunological indicators, especially in patients with established CVDs, have a high prognostic value, whereas biochemical markers are informative and accessible tools to assess aging in individuals without diagnosed diseases. Thus, the obtained numerical results not only justify the choice of the mathematical model but also open up opportunities for personalised medicine and early prevention of age-associated cardio pathologies.

This study advances the current understanding of cardiovascular aging in several key ways. First, while traditional models focus primarily on clinical or lifestyle data, our approach integrates immune system markers, biochemical indicators and socio-behavioural variables, highlighting the multifactorial nature of biological aging. This multimodal structure reflects the systemic interactions that underlie cardiovascular decline more comprehensively than existing models.

Second, most previous studies apply cross-sectional data and assume static risk. In contrast, we implemented dynamic modelling via numerical solutions of differential equations (e.g., Runge–Kutta), allowing us to simulate the progression of aging over time, a rarely addressed gap in predictive studies on cardiovascular aging.

Finally, by identifying immune aging markers (e.g., CD14, HLA-DR) as early predictors—even before clinical CVD manifestation—the model contributes to preventive medicine and personalised risk stratification, which remains a critical gap in routine cardiovascular assessments.

The proposed mathematical model was validated using real clinical data obtained from a cohort of 52 patients aged 60 years and above. The data included immunological markers (e.g., CD14, HLA-DR), biochemical indicators (e.g., NT-proBNP, SOD) and socio-behavioural factors (e.g., BMI, education, physical activity), collected under standardised protocols.

The numerical solution of the model was compared to an analytical reference derived by integrating the governing differential equation (Equation (2)). This allowed for the quantification of absolute errors using different numerical methods (Runge–Kutta, Adams–Bashforth, backward Euler), with Runge–Kutta demonstrating the lowest deviation (Table 16 and Table 17).

To further validate predictive capacity, the same dataset was used to train machine learning classifiers, including random forest, k-NN, logistic regression and XGBoost. These were evaluated using 5-fold cross-validation, with performance measured via accuracy and AUC.

Similarly, CD14, a monocyte/macrophage marker involved in innate immunity, showed significant associations with post-infarction cardiosclerosis (41%) and coronary heart disease (30%), reinforcing its role in atherosclerosis and post-necrotic remodelling, as also noted by Moore et al. (2013) [6].

Markers such as CD16 and CD56, which are involved in natural killer (NK) cell-mediated cytotoxicity, exhibited notable correlations with blood pressure and education level, indicating potential links between the social determinants of health and immune activation, a relationship increasingly reported in population-level studies [9,16].

Taken together, these findings support the validity of using immunological biomarkers as early indicators of cardiovascular aging and underscore the biological plausibility of the mathematical correlations captured in our model.

Ultimately, the model could support personalised preventive strategies and individualised patient counselling, helping delay cardiovascular deterioration through targeted interventions.

## 5. Conclusions

Correlation matrices with socio-behavioural factors were constructed on the basis of the conducted experiment on immunological and biochemical biomarkers. A mathematical model specifying the level of parameter interaction for predicting cardiovascular aging was constructed. The two problems considered were the effects of immunological and biochemical biomarkers on premature aging. The cases for all patients, without CVD, with CVD and patients without CVD were investigated separately. A method for numerical implementation of mathematical modelling was selected. The results showed that of the case studied, immunological data with SWDs showed the best results for detecting premature cardiovascular aging. And biochemical markers were effective for assessing the risk of premature aging in the absence of CVD.

A comparative analysis of the accuracy of numerical methods showed the advantage of the Runge–Kutta method of the 4th order, providing minimal deviations from the exact analytical solution for all three cases studied. For example, for patients with CVD, when immunological indices were used, the error was less than 0.000005, whereas when the Adams–Bashforth method was used, it exceeded 0.0045. A similar trend was observed for modelling using biochemical data. These results demonstrate the stability and efficiency of the chosen numerical approach for building a mathematical model of biological aging. HLA-DR (50%), CD14 (41%) and CD16 (38%) were the most significant interactions.

The body mass index also correlated with placental growth factor (37%). Glomerular filtration rate (GFR) and cystatin C, showed a positive correlation with physical activity (47%), indicating better overall health in more active patients. And, superoxide dismutase (SOD), an enzyme that protects cells from oxidative stress, showed a negative correlation with physical activity at 48%, indicating a decrease in antioxidant activity with increased metabolic load.

Thus, the integration of immunological and biochemical parameters with socio-behavioural characteristics allowed for the construction of a highly accurate model for assessing the risk of premature cardiovascular aging. Immunological markers showed the highest sensitivity in the population with established diagnoses, while biochemical markers showed the best predictive ability in the total sample. This emphasises the need for a differentiated approach when selecting diagnostic panels depending on the clinical status of the patient.

Future work should focus on validating the model in external clinical settings, and developing an interactive clinical application to support risk stratification and early intervention planning.

## Figures and Tables

**Table 1:** Immunological biomarkers.

Parameter	Description
*CD59 (Protectin)*	A protein that attaches to the membrane of cells and protects them from damage by the immune system by preventing complement activation.
*CD16 (FCγRIII)*	Receptor on the surface of some immune cells such as neutrophils, natural killer cells (NK cells) and macrophages.
*Interleukin 10 (IL-10)*	An anti-inflammatory cytokine that suppresses the inflammatory response and regulates immune system activity, preventing excessive inflammation.
*CD14*	Receptor that is found on the surface of monocytes and macrophages.
*Placenta Growth Factor (PGF)*	Placental growth factor associated with vascular development.
*CD19*	A marker found on B-lymphocytes.
*Tumour Necrosis Factor (TNF)*	A cytokine that plays an important role in inflammation.
*Vascular Endothelial Growth Factor (VEGF)*	A growth factor that stimulates the formation of new blood vessels (angiogenesis).
*CD56*	A marker found on the surface of natural killer (NK) cells.
*Perforin*	A protein that is secreted by cytotoxic T lymphocytes and NK cells to kill infected cells.
*Granulocyte–Macrophage Colony-Stimulating Factor (GM-CSF)*	A cytokine that stimulates the formation and differentiation of granulocytes and macrophages.
*CD8*	A marker present on cytotoxic T lymphocytes, which play an important role in destroying virus-infected and tumour cells.
*HLA-DR*	Class II major histocompatibility complex (MHC II) molecule found on antigen-presenting cells.
*CD95 (Fas)*	A receptor involved in the process of apoptosis.
*CD4*	A marker found on the surface of T-helper cells that coordinate the immune response.

**Table 2:** Biochemical biomarkers.

Parameter	Norm	Description
*Albumin (ALB)*	38.00–50.00 g/L	A protein synthesised in the liver. Albumin levels in the blood decrease with age. Decreased albumin levels are an independent risk factor for the development of cardiovascular disease. Hypoalbuminaemia is a precursor to an increased risk of myocardial infarctions and strokes.
*C-Reactive Protein (CRP)*	<5 mg/L	CRP levels in the blood may be an indicator of chronic inflammation.
*XЭ (ChE)*	4260.00–11,250.00 u/L	An enzyme involved in the metabolism of choline.
*NT-proBNP*	0.00–450.00 pg/mL	Natriuretic peptide associated with cardiac workload.
*Cystatin C (CTT)*	from 21 years old: 0.47–1.09 mg/L;	High cystatin C levels are associated with an increased risk of all-cause mortality and cardiovascular disease in the elderly.
*Glomerular Filtration Rate (GFR)*	>60 mL/min/1.73 m^2^	A measure of kidney function, calculated from creatinine, cystatin C and other markers.
*Superoxide Dismutase (SOD)*	164.00–240.00 u/mL	An enzyme that protects cells from oxidative stress. Mitochondrial SOD deficiency can lead to accelerated cell aging and impaired cell function.

**Table 3:** Clinical and social biomarkers.

Parameter	Description
*Smoking (S)*	One of the major risk factors for cardiovascular disease.
*Alcohol (A)*	One of the major risk factors for cardiovascular disease.
*Acute Cerebral Circulation Disorder (ACVD)*	Stroke rate.
*Physical Activity (PhA)*	An important factor in the prevention of cardiovascular disease.
*Education (E)*	Social factor affecting health through lifestyle.
*AB-Block (AB)*	Impaired conduction of electrical impulses between the atria and ventricles of the heart through the atrioventricular (AV) node.
*Atrial Fibrillation (AF)*	Heart rhythm disturbance.
*Coronary Heart Disease (CHD)*	A chronic condition associated with inadequate blood supply to the heart.
*Body Mass Index (BMI)*	Indicates the weight category (underweight, normal, overweight, obese).
*Postinfarction Cardiosclerosis (PICS)*	Chronic condition after myocardial infarction.
*Stent (CABG/stent)*	Indices of surgical interventions to restore blood supply to the heart.
*Diabetes Mellitus (DM)*	Is a chronic metabolic disease involving glucose metabolism.
*Chronic Heart Failure (CHF)*	Is a condition in which the heart is unable to pump enough blood to meet the body’s needs.
*Cerebrovascular Diseases (CVD)*	Is a group of diseases that affect the heart and blood vessels.
*Arterial Hypertension (AH)*	Arterial Hypertension.

**Table 4:** High correlation coefficients for all patients according to immunological biomarkers (Case A of Problem 1).

Case	Coefficient	Value
Case A of Problem 1	$a_{1}$	0.39
	$a_{2}$	0.37
	$a_{3}$	0.36
	$a_{4}$	0.32
	$a_{5}$	0.29
	$a_{6}$	0.3

**Table 5:** Parameters of a mathematical model of premature aging of all patients according to immunological biomarkers (Case A of Problem 1).

Case	Kind of Parameter	Parameter	Description
Case A of Problem 1	Social	$x_{1}$	BMI
		$x_{2}$	PICS
		$x_{3}$	BMI
		$x_{4}$	E
		$x_{5}$	BMI
		$x_{6}$	CHD
	Immunological	$y_{1}$	HLA.DR
		$y_{2}$	CD14
		$y_{3}$	IPGF
		$y_{4}$	CD59
		$y_{5}$	CD14
		$y_{6}$	CD14

**Table 6:** High correlation coefficients for patients with CVD according to immunological biomarkers (Case B of Problem 1).

Case	Coefficient	Value
Case B of Problem 1	$a_{1}$	0.5
	$a_{2}$	0.43
	$a_{3}$	0.41
	$a_{4}$	0.38
	$a_{5}$	0.38
	$a_{6}$	0.37

**Table 7:** Parameters of a mathematical model of premature aging of patients with CVD according to immunological biomarkers (Case B of Problem 1).

Case	Kind of Parameter	Parameter	Description
Case B of Problem 1	Social	$x_{1}$	BMI
		$x_{2}$	E
		$x_{3}$	PICS
		$x_{4}$	E
		$x_{5}$	AH
		$x_{6}$	CHD
	Immunological	$y_{1}$	HLA.DR
		$y_{2}$	HLA.DR
		$y_{3}$	CD14
		$y_{4}$	CD56
		$y_{5}$	CD16
		$y_{6}$	IPGF

**Table 8:** High correlation coefficients for patients without CVD according to immunological biomarkers (Case C of Problem 1).

Case	Coefficient	Value
Case C of Problem 1	$a_{1}$	0.67
	$a_{2}$	0.43
	$a_{3}$	0.43
	$a_{4}$	0.42
	$a_{5}$	0.39
	$a_{6}$	0.37

**Table 9:** Parameters of a mathematical model of the premature aging of patients without CVD according to immunological biomarkers (Case C of Problem 1).

Case	Kind of Parameter	Parameter	Description
Case C of Problem 1	Social	$x_{1}$	CVD
		$x_{2}$	A
		$x_{3}$	BMI
		$x_{4}$	PhA
		$x_{5}$	E
		$x_{6}$	PhA
	Immunological	$y_{1}$	II10
		$y_{2}$	CD95
		$y_{3}$	ICD95
		$y_{4}$	CD59
		$y_{5}$	CD59
		$y_{6}$	IPGF

**Table 10:** High correlation coefficients for all patients according to biochemical biomarkers (Case A of Problem 2).

Case	Coefficient	Value
Case A of Problem 2	$a_{1}$	0.43
	$a_{2}$	0.41
	$a_{3}$	0.38
	$a_{4}$	0.33
	$a_{5}$	0.29
	$a_{6}$	0.31

**Table 11:** Parameters of a mathematical model of the premature aging of all patients according to biochemical biomarkers (Case A of Problem 2).

Case	Kind of Parameter	Parameter	Description
Case A of Problem 2	Social	$x_{1}$	CVD
		$x_{2}$	ACVD
		$x_{3}$	BMI
		$x_{4}$	CVD
		$x_{5}$	AH
		$x_{6}$	ACVD
	Biochemical	$y_{1}$	CTT
		$y_{2}$	ALB
		$y_{3}$	GFR
		$y_{4}$	SOD
		$y_{5}$	SOD
		$y_{6}$	GFR

**Table 12:** High correlation coefficients for patients with CVD according to biochemical biomarkers (Case B of Problem 2).

Case	Coefficient	Value
Case B of Problem 2	$a_{1}$	0.47
	$a_{2}$	0.43
	$a_{3}$	0.33
	$a_{4}$	0.3
	$a_{5}$	0.24
	$a_{6}$	0.24

**Table 13:** Parameters of a mathematical model of the premature aging of patients with CVD according to biochemical biomarkers (Case B of Problem 2).

Case	Kind of Parameter	Parameter	Description
Case B of Problem 2	Social	$x_{1}$	PhA
		$x_{2}$	PhA
		$x_{3}$	ACVD
		$x_{4}$	AB
		$x_{5}$	S
		$x_{6}$	stent
	Biochemical	$y_{1}$	GFR
		$y_{2}$	ChE
		$y_{3}$	CRP
		$y_{4}$	*ChE*
		$y_{5}$	CTT
		$y_{6}$	ChE

**Table 14:** High correlation coefficients for patients without CVD according to biochemical biomarkers (Case C of Problem 2).

Case	Coefficient	Value
Case C of Problem 2	$a_{1}$	0.55
	$a_{2}$	0.48
	$a_{3}$	0.36
	$a_{4}$	0.36
	$a_{5}$	0.29
	$a_{6}$	0.24

**Table 15:** Parameters of a mathematical model of the premature aging of patients without CVD according to biochemical biomarkers (Case C of Problem 2).

Case	Kind of Parameter	Parameter	Description
Case C of Problem 2	Social	$x_{1}$	BMI
		$x_{2}$	BMI
		$x_{3}$	E
		$x_{4}$	PhA
		$x_{5}$	CVD
		$x_{6}$	A
	Biochemical	$y_{1}$	ALB
		$y_{2}$	GFR
		$y_{3}$	SOD
		$y_{4}$	SOD
		$y_{5}$	SOD
		$y_{6}$	CTT

**Table 16:** Order of accuracy of numerical methods for modelling premature aging with immunological biomarkers (Problem 1).

Problem	Solution Method	Order of Method	Case	Maximum Error with the Exact Solution $\max\left|x_{i}^{\mathrm{exact}}-x_{i}^{\mathrm{method}}\right|$
Problem 1	Runge–Kutta	$O({∆x}^{4})$	Case A	0.000007536924
			Case B	0.000004918764
			Case C	0.00000284855
	Backward Euler	$O({∆x}^{2})$	Case A	0.004240115
			Case B	0.00304603
			Case C	0.002952076
	Adams–Bashforth	$O\left(∆x\right)$	Case A	0.006360173
			Case B	0.004569044
			Case C	0.004428114

**Table 17:** Order of accuracy of numerical methods for modelling premature aging with biochemical biomarkers (Problem 2).

Problem	Solution Method	Order of Method	Case	Maximum Error with the Exact Solution $\max\left|x_{i}^{\mathrm{exact}}-x_{i}^{\mathrm{method}}\right|$
Problem 2	Runge–Kutta	$O({∆x}^{4})$	Case A	0.000004710849
			Case B	0.00001958067
			Case C	0.000002147958
	Backward Euler	$O({∆x}^{2})$	Case A	0.002740348
			Case B	0.003500058
			Case C	0.004239759
	Adam–Bashforth	$O\left(∆x\right)$	Case A	0.004110521
			Case B	0.005250088
			Case C	0.006359639

**Table 18:** Parameters of modelling.

Determination	Value
Time step, ∆t	0.01
Grid step ∆x	0.001
Number of iterations, N	1000
Calculation interval, [*a*;*b*]	[0;1]

## Data Availability

The presented data in this study are not publicly available due to ongoing research, ethical restrictions, and the need to protect the confidentiality of study participants.

## References

[1] World Health Organization (2023). Cardiovascular Diseases (CVDs).

[2] D’Agostino R.B., Vasan R.S., Pencina M.J., Wolf P.A., Cobain M., Massaro J.M., Kannel W.B. (2008). General cardiovascular risk profile for use in primary care: The Framingham Heart Study. Circulation.

[3] Libby P. (2021). The changing landscape of atherosclerosis. Nature.

[4] Ridker P.M. (2016). From C-reactive protein to interleukin-6 to interleukin-1: Moving upstream to identify novel targets for atheroprotection. Circ. Res..

[5] Hansson G.K., Hermansson A. (2011). The immune system in atherosclerosis. Nat. Immunol..

[6] Moore K.J., Sheedy F.J., Fisher E.A. (2013). Macrophages in atherosclerosis: A dynamic balance. Nat. Rev. Immunol..

[7] Maisel A.S., Krishnaswamy P., Nowak R.M., McCord J., Hollander J.E., Duc P., Omland T. (2002). Rapid measurement of B-type natriuretic peptide in the emergency diagnosis of heart failure. N. Engl. J. Med..

[8] Ix J.H., Shlipak M.G. (2007). Cystatin C and prognosis in cardiovascular disease: A recent meta-analysis. J. Am. Coll. Cardiol..

[9] Marmot M., Friel S., Bell R., Houweling T.A.J., Taylor S. (2008). Closing the gap in a generation: Health equity through action on the social determinants of health. Lancet.

[10] Butcher J.C. (2016). Numerical Methods for Ordinary Differential Equations.

[11] Hairer E., Wanner G. (2010). Solving Ordinary Differential Equations II: Stiff and Differential-Algebraic Problems.

[12] Bafei S.E.C., Shen C. (2023). Biomarkers Selection and Mathematical Modeling in Biological Age Estimation. npj Aging.

[13] Libert S., Chekholko A., Kenyon C. (2024). A Mathematical Model That Predicts Human Biological Age from Physiological Traits Identifies Environmental and Genetic Factors That Influence Aging. Comput. Syst. Biol..

[14] Suleimenova M., Abzaliyev K., Bugibayeva A., Abzaliyeva S., Sundetova D., Kurmanova A., Karashash A., Nurbakyt A., Jardemova K., Nurbakyt A. (2024). Application of Machine Learning in Identifying Premature Aging. Arch. Gerontol. Geriatr. Res..

[15] Abzaliyev K., Suleimenova M., Chen S., Mansurova M., Abzaliyeva S., Manapova A., Kurmanova A., Bugibayeva A., Sundetova D., Bitemirova R. (2025). Predicting Cardiovascular Aging Risk Based on Clinical Data Through the Integration of Mathematical Modeling and Machine Learning. Appl. Sci..

[16] Cevirgel A., Shetty S.A., Vos M., Nanlohy N.M., Beckers L., Bijvank E., Rots N., van Beek J., Buisman A.-M., van Baarle D. (2022). Identification of Aging-Associated Immunotypes and Immune Stability as Indicators of Post-Vaccination Immune Activation. Aging Cell.

[17] Levochkina E. (2023). Immunological Markers in the Diagnosis of Cardiovascular Diseases: Prospects and Forecasts. CyberLeninka. https://cyberleninka.ru/article/n/immunologicheskie-markery-v-diagnostike-serdechno-sosudistyh-zabolevaniy-perspektivy-i-prognozy/viewer.

[18] Ravera S., Vigliarolo T., Bruno S., Morandi F., Marimpietri D., Sabatini F., Dagnino M., Petretto A., Bartolucci M., Muraca M. (2021). Identification of Biochemical and Molecular Markers of Early Aging in Childhood Cancer Survivors. Cancers.

[19] Mao C., Yuan J.Q., Lv Y.B., Gao X., Yin Z.X., Kraus V.B., Luo J.S., Chei C.L., Matchar D.B., Zeng Y. (2019). Associations between superoxide dismutase, malondialdehyde and all-cause mortality in older adults: A community-based cohort study. BMC Geriatr..

[20] Sarnak M.J., Katz R., Fried L.F., Siscovick D., Kestenbaum B., Seliger S., Rifkin D., Tracy R., Newman A.B., Shlipak M.G. (2008). Cystatin C and aging success. Arch. Intern. Med..

[21] Yang H., Liao Z., Zhou Y., Gao Z., Mao Y. (2024). Non-linear relationship of serum albumin-to-globulin ratio and cognitive function in American older people: A cross-sectional national health and nutrition examination survey 2011–2014 (NHANES) study. Front. Public Health.

[22] Muscari A., Bianchi G., Forti P., Magalotti D., Pandolfi P., Zoli M., Pianoro Study Group (2021). N-terminal pro B-type natriuretic peptide (NT-proBNP): A possible surrogate of biological age in the elderly people. Geroscience.

[23] Sayed-Ahmed N.M.Z., Hossain M.A., Limkar S., El-Bahkiry H.S., Alam N., Amin S.T. (2024). Mathematical Modelling and Deep Learning Techniques for Predicting Cardiovascular Disease. Panam. Math. J..

[24] Suleimenova M., Abzaliyev K., Mansurova M., Abzaliyeva S., Kurmanova A., Tokhtakulinova G., Bugibayeva A., Sundetova D., Abdykassymova M., Sagalbayeva U. (2025). A Predictive Model of Cardiovascular Aging by Clinical and Immunological Markers Using Machine Learning. Diagnostics.

[25] Abhishek, Bhagat H.V., Singh M. A Machine Learning Model for the Early Prediction of Cardiovascular Disease in Patients. Proceedings of the 2023 Second International Conference on Advances in Computational Intelligence and Communication (ICACIC).

